# Reviewer acknowledgement 2015

**DOI:** 10.1186/s13068-016-0453-x

**Published:** 2016-02-26

**Authors:** James du Preez, Michael Himmel, Debra Mohnen, Charles Wyman

**Affiliations:** Department of Microbial, Biochemical and Food Biotechnology, University of the Free State, Bloemfontein, South Africa; Bioscience Center, National Renewable Energy Laboratory, Golden, CO USA; Department of Biochemistry and Molecular Biology, Complex Carbohydrate Research Center, Athens, GA USA; Department of Microbial, University of California, Riverside, Riverside, CA USA; Department of Chemical and Environmental Engineering, University of California, Riverside, Riverside, CA USA

## Contributing reviewers

*Biotechnology for Biofuels* would like to thank all reviewers who have contributed to the journal in 2015.

**Gabriel Acien**

Spain

**Sunil Adav**

Singapore

**Felix Adom**

USA

**Mukund Adsul**

India

**George Aggelis**

Greece

**Deepti Agrawal**

India

**Gennaro Agrimi**

Italy

**André Aguiar**

Brazil

**Joong-Hoon Ahn**

South Korea

**M Kalim Akhtar**

UK

**Gabriela Alfaro-Espinoza**

Germany

**Benjamin Allardyce**

Australia

**Grant Allen**

Canada

**Doug Allen**

USA

**João R M Almeida**

Brazil

**David Alonso**

USA

**Hal Alper**

USA

**Alberto Amaretti**

Italy

**Diletta Ami**

Italy

**Pratheep Annamalai**

Australia

**Jens Appel**

Germany

**Klaus-J Appenroth**

Germany

**Munehito Arai**

Japan

**Patricia Armshaw**

Ireland

**Vincent Arondel**

France

**Mikko Arvas**

Finland

**Mark Peter Ashe**

UK

**Arun Athmanathan**

USA

**Melani Atmodjo**

USA

**Shota Atsumi**

USA

**Manfred Auer**

USA

**Fengwu Bai**

China

**John Baker**

USA

**Venkatesh Balan**

USA

**Jie Bao**

China

**Xiaoming Bao**

China

**Luis Barahona-Perez**

Mexico

**Abdellatif Barakat**

France

**Brian Barr**

USA

**Laura Bartley**

USA

**Mirko Basen**

Germany

**Vinod Bathula**

USA

**Stefan Bauer**

USA

**Gregg Beckham**

USA

**Matthew Bennett**

USA

**Alex Berlin**

USA

**Rafael Bernardi**

USA

**Nicolas Bernet**

France

**Jean-Guy Berrin**

France

**Gwénaëlle Bestel-Corre**

France

**Maurizio Bettiga**

Sweden

**Fabrizio Bezzo**

Italy

**Samarthya Bhagia**

USA

**Aditya Bhalla**

USA

**Hem Bhandari**

USA

**Peter Biely**

Slovakia

**Damla Bilgin**

USA

**D Biria**

Iran

**Kateřina Bišová**

Czech Republic

**Rajib Biswas**

USA

**Jeffrey Blanchard**

USA

**Hans Blaschek**

USA

**Bastin Blombach**

Germany

**Sara Blumer-Schuette**

USA

**Wout Boerjan**

Belgium

**Rafal Bogel-Lukasik**

Portugal

**Gregory Bokinsky**

The Netherlands

**Silvia Bolado**

Spain

**Eckhard Boles**

Germany

**Antonio Bonomi**

Brazil

**Mallika Boonmee**

Thailand

**Alasdair Boraston**

Canada

**Florent Bouxin**

UK

**Paola Branduardi**

Italy

**Tore Brembu**

Norway

**Andreas Bremges**

Germany

**Michel Brienzo**

Brazil

**Patrick Brown**

USA

**Steven Brown**

USA

**T Bruck**

Germany

**Mahesh Bule**

USA

**Renata Bura**

USA

**Michael Burkart**

USA

**Gloria Burow**

USA

**Kara Cafferty**

USA

**Zhen Cai**

China

**Zeynep Petek Cakar**

Turkey

**Jesus Campos-García**

Mexico

**Thomas Canam**

USA

**Isaac Cann**

USA

**Danilo A Cantero**

Spain

**Yi Cao**

China

**Marta Carballa**

Spain

**Magnus Carlquist**

Sweden

**Ross Carlson**

USA

**Alberta Carpenter**

USA

**Rosario Castillo**

Chile

**Jamie Cate**

USA

**Ewelina Celinska**

Poland

**Verawat Champreda**

Thailand

**Richard Chandra**

Canada

**Matthew Chang**

Singapore

**Jo-Shu Chang**

Taiwan

**Varodom Charoensawan**

Thailand

**Afroditi Chatzifragkou**

UK

**Benjamas Cheirsilp**

Thailand

**Shreeshivadasan Chelliapan**

Malaysia

**Guo-Qiang Chen**

China

**Hongge Chen**

China

**Hongzhang Chen**

China

**Xin-de Chen**

China

**Yinguang Chen**

China

**Chun-Yen Chen**

Taiwan

**Bor-Yann Chen**

Taiwan

**Xiaowen Chen**

USA

**Gang Cheng**

China

**Yu-Shen Cheng**

Taiwan

**David Chiaramonti**

Italy

**C Perry Chou**

Canada

**Te-Jin Chow**

Taiwan

**Soumitra Chowdhury**

Germany

**Paul Christakopoulos**

Sweden

**Kung-Hui Chu**

USA

**Christopher Chuck**

UK

**Marek Cieplak**

Poland

**Anthony Clarke**

Canada

**James Coker**

USA

**Andre Coleman**

USA

**Ulrich Commandeur**

Germany

**Cristobal Corpas**

Spain

**Aline Costa**

Brazil

**Qiu Cui**

China

**Ting Cui**

China

**Yi Cui**

China

**Dan Cullen**

USA

**Etienne Dague**

France

**Junbiao Dai**

China

**Jean-Marc Daran**

The Netherlands

**Maurycy Daroch**

China

**Debabrata Das**

India

**Mark Davis**

USA

**Brian Davison**

USA

**Eduardo R de Azevedo**

Brazil

**Heizir De Castro**

Brazil

**Riaan de Haan**

South Africa

**Marcos Antonio De Morais Jr.**

Brazil

**Wanderley de Souza**

Brazil

**Ronald de Vries**

The Netherlands

**Steve Decker**

USA

**Cristina Del Bianco**

Italy

**Luis Del Rio**

Canada

**Deborah Delmer**

USA

**Jaclyn DeMartini**

USA

**Marie Demuez**

Spain

**Santino Di Berardino**

Portugal

**Bruce Dien**

USA

**Maria Dimarogona**

Sweden

**Shi-You Ding**

USA

**Elio Dinuccio**

Italy

**Jayna Ditty**

USA

**Lucilia Domingues**

Portugal

**Bryon Donohoe**

USA

**Carl Douglas**

Canada

**Arnold Driessen**

The Netherlands

**Dorra Driss**

Tunisia

**Irina Druzhinina**

Austria

**Guocheng Du**

China

**Zhuwei Du**

China

**Kow Jen Duan**

Taiwan

**Tim Dumonceaux**

Canada

**Laurence Dumon-Seignovert**

France

**Jennifer Dunn**

USA

**Robert Dunn**

USA

**Simon Dusseau**

France

**Paul Dyer**

UK

**Armin Ehrenreich**

Germany

**Vincent Eijsink**

Norway

**W Eisenreich**

Germany

**Thomas Elder**

USA

**James Elkins**

USA

**José Empis**

Portugal

**Borbala Erdei**

Sweden

**Jaime Eyzaguirre**

Chile

**Thaddeus Ezeji**

USA

**Zhiliang Fan**

USA

**Xu Fang**

China

**Zhen Fang**

China

**Jianmin Fang**

UK

**Vincenza Faraco**

Italy

**Qiang Fei**

USA

**Claus Felby**

Denmark

**Jiaxun Feng**

China

**Francisco Jesus Fernandez Morales**

Spain

**Andre Ferraz**

Brazil

**Antônio Djalma Ferraz Júnior**

Brazil

**Marcia Ferreira**

Brazil

**Viridiana Ferreira-Leitão**

Brazil

**Manuel Ferrer**

Spain

**Henri-Pierre Fierobe**

France

**Francisco Florencio**

Spain

**César Fonseca**

Portugal

**Marcus Foston**

USA

**Eric Fouilland**

France

**Brian Fox**

USA

**Marco Fraaije**

The Netherlands

**Mary Ann Franden**

USA

**Stefano Freguia**

Australia

**Jesper Frickmann**

USA

**Pengcheng Fu**

China

**Zui Fujimoto**

Japan

**Papa Gabriella**

USA

**Mats Galbe**

Sweden

**Anna Maria Galletti**

Italy

**Xue Gao**

USA

**María del Prado García Aparicio**

South Africa

**Xin Ge**

USA

**Xumeng Ge**

USA

**Anli Geng**

Singapore

**Donna Gibson**

USA

**Glenda E Gillaspy**

USA

**S Gkelis**

Greece

**N Louise Glass**

USA

**D V Gokhale**

India

**Gustavo Goldman**

Brazil

**Paula Goncalves**

Portugal

**Cristina Gonzalez**

Spain

**Maria I González Siso**

Spain

**K Gopalakrishnan**

India

**Johann Gorgens**

South Africa

**Steven Gorsich**

USA

**Ester Gouveia**

Brazil

**Bernhard Grimm**

Germany

**Dominique Grizeau**

France

**Melanie Grogger**

USA

**Amy Grunden**

USA

**Yang Gu**

China

**Steinn Gudmundsson**

Iceland

**Jianhua Guo**

China

**Wan-Qian Guo**

China

**Miao Guo**

UK

**Vijai Kumar Gupta**

Ireland

**Raghu Gurram**

USA

**Adam Guss**

USA

**Gisela Guthausen**

Germany

**Ji-Sook Hahn**

South Korea

**Patrick Hallenbeck**

Canada

**Jerome Hamelin**

France

**Sung Ok Han**

South Korea

**Taizo Hanai**

Japan

**Henrik Hansson**

Sweden

**Valerie Harmon**

USA

**Annele Hatakka**

Finland

**Samuel Hazen**

USA

**Ning He**

China

**Pinjing He**

China

**Zhen He**

USA

**Ronald Hector**

USA

**Kai Heimel**

Germany

**Klaas Hellingwerf**

The Netherlands

**Bernard Henrissat**

France

**Michael Henson**

USA

**Pérez Herminia Inés**

Mexico

**Mark Hildebrand**

USA

**Shih-Hsin Ho**

Taiwan

**David Hodge**

USA

**Mitsuo Horita**

Japan

**Svein Jarle Horn**

Norway

**Jinguang Hu**

Canada

**Hanhua Hu**

China

**Zhenhu Hu**

China

**He Huang**

China

**Chi-Yu Huang**

Taiwan

**Fang Huang**

USA

**Paul Hudson**

Sweden

**Michael Huesemann**

USA

**Steve Hutcheson**

USA

**David Ibarra**

Spain

**Marja Ilmen**

Finland

**Augusta Isaac**

Brazil

**Takuro Ito**

Japan

**Christa Ivanova**

France

**Makio Iwahashi**

Japan

**Javier Izquierdo**

USA

**Jungho Jae**

USA

**Nikhil Jaikumar**

USA

**Yu-Sin Jang**

South Korea

**Kaemwich Jantama**

Thailand

**Grzegorz Janusz**

Poland

**Cy Jeffries**

Germany

**Tina Jeoh**

USA

**Byong-Hun Jeon**

South Korea

**Min Jiang**

China

**Rongrong Jiang**

Singapore

**Diego Jimenez**

The Netherlands

**Eon Seon Jin**

South Korea

**Mingjie Jin**

USA

**Katja Johansen**

Sweden

**Jane Johnson**

USA

**Patrik Jones**

Finland

**Ann-Sofi Jonsson**

Sweden

**Chijioke Joshua**

USA

**Yi-Je Juang**

Taiwan

**Gyoo Yeol Jung**

South Korea

**David Kaftan**

Czech Republic

**Katy Kao**

USA

**Keikhosro Karimi**

Iran

**Susan Karp**

Brazil

**Rui Katahira**

USA

**Katariina Kemppainen**

Finland

**William Kenealy**

USA

**Eduard Kerkhoven**

Sweden

**Byung-Gee Kim**

South Korea

**Dong-Hoon Kim**

South Korea

**K Thomas Klasson**

USA

**Sabine Kleinsteuber**

Germany

**Mario Klimacek**

Austria

**Eric Knoshaug**

USA

**Mattheos Koffas**

USA

**Magdalena Kolbusz**

Canada

**Akihiko Kondo**

Japan

**Ioannis Kookos**

Greece

**Maarten Kootstra**

The Netherlands

**Takuya Koseki**

Japan

**Kornel L Kovacs**

Hungary

**Krisztina Kovacs**

Sweden

**Emma Kreuger**

Sweden

**Malgorzata Krzywonos**

Poland

**Ajay Kumar**

India

**Ravindra Kumar**

India

**Shashi Kumar**

India

**Adepukiran Kumar**

USA

**Rajeev Kumar**

USA

**Masao Kunioka**

Japan

**Rodrigo Labatut**

USA

**Paulo Lacava**

Brazil

**Michael Ladisch**

USA

**Cecilia Laluce**

Brazil

**Ethan Lan**

Taiwan

**Siegmund Lang**

Germany

**Lene Lange**

Denmark

**Lakkana Laopaiboon**

Thailand

**Edmundo Larenas**

USA

**Christer Larsson**

Sweden

**Chyi-How Lay**

Taiwan

**Zbigniew Lazar**

France

**Changsoo Lee**

South Korea

**Jin-Woo Lee**

South Korea

**Jung-Kul Lee**

South Korea

**Cheng-Kang Lee**

Taiwan

**Sang Hun Lee**

USA

**Y Y Lee**

USA

**Gustavo Leite**

Canada

**Patricia Inés Leonardi**

Argentina

**Susanna Leong**

Singapore

**Fuli Li**

China

**Hong-Ye Li**

China

**Jianzheng Li**

China

**Shengying Li**

China

**Shizhong Li**

China

**Yin Li**

China

**Si-Yu Li**

Taiwan

**Chenlin Li**

USA

**Hongjia Li**

USA

**Shundai Li**

USA

**Xiaobo Li**

USA

**Jiazhang Lian**

USA

**Jieni Lian**

USA

**James Liao**

USA

**Yonghua Li-Beisson**

France

**Gunnar Liden**

Sweden

**Carmen Limon**

Spain

**Shanzhi Lin**

China

**Chih-Sheng Lin**

Taiwan

**Jeff Linger**

USA

**Dehua liu**

China

**Jianhua Liu**

China

**Tiangang Liu**

China

**Weifeng Liu**

China

**Zhidan Liu**

China

**Dajiang Liu**

USA

**Fang Liu**

USA

**Hong Liu**

USA

**Xiaoguang Margaret Liu**

USA

**Yan Liu**

USA

**Adrian Lopez Garcia de Lomana**

USA

**Isabel M Lopez-Lara**

Mexico

**Etienne Low-Decarie**

UK

**Roland Ludwig**

Austria

**Taina Lundell**

Finland

**Xiaolan Luo**

USA

**Jason Lupoi**

USA

**Francisco Luque**

Spain

**Jeremy Luterbacher**

Switzerland

**Gary Lye**

UK

**Lee Lynd**

USA

**Kesen Ma**

Canada

**Cuiqing Ma**

China

**Warren Mabee**

Canada

**Robert Mach**

Austria

**Astrid Rosa Mach-Aigner**

Austria

**Stefano Macrelli**

Sweden

**Miia Mäkelä**

Finland

**Francisco Manzano-Agugliaro**

Spain

**Eric Marechal**

France

**Giorgos Markou**

Greece

**Isabel Marques**

Portugal

**Paula Marques**

Portugal

**Darren Martin**

Australia

**Alfredo Martinez**

Mexico

**Pablo Martín-Ramos**

Spain

**Christopher Marx**

USA

**Emma Master**

Canada

**Cesar Mateo**

Spain

**Leonidas Matsakas**

Sweden

**Harold D May**

USA

**John McBride**

USA

**Mark McBride**

USA

**Armando McDonald**

USA

**Jon McKechnie**

UK

**Lauren McKee**

Sweden

**Sebastian Meier**

Denmark

**Richard Meilan**

USA

**Anastasios Melis**

USA

**Simona Menardo**

Italy

**Anne S Meyer**

Denmark

**Christian Meyer**

France

**Isabelle Meynial-Salles**

France

**Krystian Miazek**

Belgium

**Todd Michael**

USA

**Michele Michelin**

Brazil

**Célia Miguel**

Portugal

**Michel Minier**

France

**Saroj Mishra**

India

**Itzhak Mizrahi**

Israel

**Alicia Modenbach**

USA

**S Venkata Mohan**

India

**Vijayanand Moholkar**

India

**Cedric Montanier**

France

**Stephen Moose**

USA

**Sarah Moraïs**

Israel

**Antonio Moreno**

Sweden

**John Morgan**

USA

**Haruhide Mori**

Japan

**Tomas Morosinotto**

Italy

**William Morrow**

USA

**Nathan Mosier**

USA

**Yang Mu**

China

**Aindrila Mukhopadhyay**

USA

**Jan Mumme**

Germany

**Andrew Munro**

UK

**Miguel C Mussati**

Argentina

**Endre Nagy**

Hungary

**Lata Nain**

India

**Shunichi Nakayama**

Japan

**Johnathan Napier**

UK

**Duangkamol Na-Ranong**

Thailand

**Helena Nevalainen**

Australia

**I-Son Ng**

Taiwan

**Jean-Marc Nicaud**

France

**Nancy Nichols**

USA

**Bernd Nidetzky**

Austria

**Alex Nielsen**

Denmark

**Sue Nokes**

USA

**Intawat Nookaew**

Sweden

**Leonel Nunes**

Portugal

**Masyuki Oda**

Japan

**Kyung Keun Oh**

South Korea

**Shigeru Okada**

Japan

**Piotr Oleskowicz-Popiel**

Poland

**Jose Miguel Oliva**

Spain

**Daniel Olson**

USA

**Michelle O’Malley**

USA

**Mislav Oreb**

Germany

**Gabriel Paes**

France

**Jose Palomo**

Spain

**Deepak Pant**

Belgium

**Seraphim Papanikolaou**

Greece

**Ioannis Papapetridis**

The Netherlands

**E Terry Papoutsakis**

USA

**Chulhwan Park**

South Korea

**Jong Moon Park**

South Korea

**Sunghoon Park**

South Korea

**Yong-Cheol Park**

South Korea

**Youn Il Park**

South Korea

**Gopal Pattanayak**

USA

**Sudhanshu Pawar**

Sweden

**Gilles Peltier**

France

**Xuya Peng**

China

**Olimpia Pepe**

Italy

**Caroline Peres**

USA

**Francisc Peter**

Romania

**Robyn Peterson**

Australia

**Loukas Petridis**

USA

**Brian Pfleger**

USA

**Julio Polaina**

Spain

**Denis Pompon**

France

**Manuel Porcar**

Spain

**Matthew Posewitz**

USA

**Justin Powlowski**

Canada

**Rolf Prade**

USA

**O Prakash**

India

**Sebastià Puig**

Spain

**Michael Pyne**

Canada

**Yinbo Qu**

China

**Arthur Ragauskas**

USA

**Christine Raines**

UK

**Karthikeyan Ramachandriya Dharman**

USA

**Parthasarathi Ramakrishnan**

USA

**Zhiming Rao**

China

**Dominik Refardt**

Switzerland

**Leticia Regueiro Abelleria**

Spain

**Amanda Reider**

USA

**Claire Remacle**

Belgium

**Nan-Qi Ren**

China

**Scott Renneckar**

Canada

**Liliane Ribeiro**

USA

**Hans Richnow**

Germany

**Oscar Rojas-Rejón**

Mexico

**Roger Ruan**

USA

**Kristine Rugele**

Latvia

**Eiji Sakuradani**

Japan

**Kourosh Salehi-Ashtiani**

UAE

**Markku Saloheimo**

Finland

**Nicholas Sandoval**

USA

**Rajesh Sani**

USA

**Ayla Santana da Silva**

Brazil

**Ilona Sarvari Horvath**

Sweden

**R Sathishkumar**

India

**Noppadon Sathitsuksanoh**

USA

**Scott Sattler**

USA

**Michael Sauer**

Austria

**Gourvendu Saxena**

Singapore

**Richard Sayre**

USA

**Peter Schaap**

The Netherlands

**Michael Scharf**

USA

**Henrik Scheller**

USA

**Robert Schmidt**

USA

**Monika Schmoll**

Austria

**Timo Schuerg**

USA

**Wolfgang Schwarz**

Germany

**Thomas Schweder**

Germany

**Paul Schweiger**

USA

**Alberto Scoma**

Belgium

**Nigel Scrutton**

UK

**Fernando Segato**

Brazil

**Gerd Seibold**

Germany

**Bernhard Seiboth**

Austria

**Jin-Ho Seo**

South Korea

**Luis Serrano**

France

**Abdelghani Sghir**

France

**K T Shanmugam**

USA

**Joe Shaw**

USA

**Yaling Shen**

China

**Claire Shen**

Taiwan

**Fei Shen**

USA

**Reshma Shetty**

USA

**Jay Shockey**

USA

**Yuval Shoham**

Israel

**Pau Loke Show**

Malaysia

**Dev Shrestha**

USA

**Pratyoosh Shukla**

India

**Verena Siewers**

Sweden

**Matti Siika-Aho**

Finland

**Roberto Silva**

Brazil

**Sang Jun Sim**

South Korea

**Blake Simmons**

USA

**Steven Singer**

USA

**Rahul Singh**

Canada

**Mika Sipponen**

Finland

**Alexandra Yu Skorokhodova**

Russia

**Nadia Skorupa Parachin**

Brazil

**Oleksandr Skyba**

Canada

**Patricia Slininger**

USA

**Steven P Smith**

Canada

**Rosario Solera**

Spain

**Yuanda Song**

China

**Philippe Soucaille**

France

**Laurence Soussan**

France

**Richard Sparling**

Canada

**Stefano Spertino**

Italy

**Fabio Squina**

Brazil

**Jerry Ståhlberg**

Sweden

**Boris Stambuck**

Brazil

**Andreas Stamer**

Switzerland

**Gregory Stephanopoulos**

USA

**Neal Stewart**

USA

**Jonathan Stickel**

USA

**Rajeev Sukumaran**

India

**Run-Cang Sun**

China

**Sandhya Swaminathan**

India

**Monika Szymanska-Chargot**

Poland

**Chika Tada**

Japan

**Yutaka Tamaru**

Japan

**Yueqin Tang**

China

**Yinjie J Tang**

USA

**Shuji Tani**

Japan

**Ralph Tanner**

USA

**Paulo Waldir Tardioli**

Brazil

**Katia Teixeira**

Brazil

**Maija Tenkanen**

Finland

**Sarah Teter**

USA

**Ralf Thauer**

Germany

**Susanne Theuerl**

Germany

**Johan Thevelein**

Belgium

**Damien Thompson**

Ireland

**Xiaofei Tian**

Canada

**Pingfang Tian**

China

**Liang Tian**

USA

**Folke Tjerneld**

Sweden

**Gaku Tokuda**

Japan

**Elia Tomas**

Spain

**Evangelos Topakas**

Greece

**Giuseppe Torzillo**

Italy

**Jennifer Town**

Canada

**Dang Thuan Tran**

Vietnam

**K Trchounian**

Armenia

**Pier-Luc Tremblay**

Denmark

**Cong Trinh**

USA

**Cong Trinh**

USA

**Shiou-Chuan Tsai**

USA

**Suguru Tsuchimoto**

Japan

**Evren Tugtas**

Turkey

**Danielle Tullman-Ercek**

USA

**Cirano Ulhoa**

Brazil

**Pilanee Vaithanomsat**

Thailand

**Priit Valjamae**

Estonia

**Richard van Kranenburg**

The Netherlands

**Ed Van Niel**

Sweden

**Jonathan Van Wagenen**

Denmark

**Willem Van Zyl**

South Africa

**Bart Vander Bruggen**

Belgium

**Alisa Vangnai**

Thailand

**Arul Mozhy Varman**

USA

**Aniko Varnai**

Norway

**Juliana Vasco**

USA

**Isabel Vasconcelos**

Portugal

**Yael Vazana**

Israel

**Wim Vermaas**

USA

**Andreas Wagner**

Austria

**Heiko Wagner**

Germany

**Aljoscha Wahl**

The Netherlands

**Keith Waldron**

UK

**Larry Walker**

USA

**Ola Wallberg**

Sweden

**Wei Wan**

USA

**Guangyi Wang**

China

**Qin-Hong Wang**

China

**Shi-An Wang**

China

**Shurong Wang**

China

**Xin Wang**

China

**Yong Wang**

China

**Ting-Fang Wang**

Taiwan

**Yi Wang**

USA

**Ethan Warner**

USA

**Kazuya Watanabe**

Japan

**Dong-Zhi Wei**

China

**Wei Wei**

China

**Yu-Hong Wei**

Taiwan

**Hui Wei**

USA

**Paul Weimer**

USA

**Noah Weiss**

Denmark

**Volker Wendisch**

Germany

**Bjorge Westereng**

Norway

**Peter Westh**

Denmark

**Janet Westpheling**

USA

**James Matthew White**

USA

**Timothy Whitehead**

USA

**Juergen Wiegel**

USA

**Zeev Wiesman**

Israel

**Christian Wilhelm**

Germany

**Cranos Williams**

USA

**David Wilson**

USA

**Flavia Vischi Winck**

Brazil

**Holzwirt Windt**

Germany

**Anders Wingren**

Sweden

**Klaus Winzer**

UK

**Litz Wobbe**

Germany

**Dominic Wong**

USA

**Guo-Jiang Wu**

China

**Jin Chuan Wu**

Singapore

**Charles Wyman**

USA

**Mo Xian**

China

**Jingli Xie**

China

**Eduardo Ximenes**

USA

**Defeng Xing**

China

**Charilaos Xiros**

Sweden

**Zhi-Long Xiu**

Canada

**Feng Xu**

China

**Jian Xu**

China

**Ping Xu**

China

**Xuan Xu**

China

**Fuqing Xu**

USA

**Chuang Xue**

China

**Song Xue**

China

**Alexander Yakunin**

Canada

**Juan Yan**

China

**Chengshi Yan**

USA

**Yajun Yan**

USA

**Sheng Yang**

China

**Quanling Yang**

Japan

**Qiang Yang**

USA

**Shang-Tian Yang**

USA

**Shihui Yang**

USA

**Shinichi Yano**

Japan

**Syed Shams Yazdani**

India

**H-W Yen**

Taiwan

**Wen Shan Yew**

Singapore

**William York**

USA

**Aiqun Yu**

Singapore

**Zhongtang Yu**

USA

**Ze-Chun Yuan**

Canada

**Tongqi Yuan**

China

**Ying-Jin Yuan**

China

**Heyang Yuan**

USA

**Junjie Yue**

China

**Fengxia Yue**

USA

**Wu Yulong**

China

**V Zachleder**

Czech Republic

**Mohd Zakaria**

Japan

**Ignacio Zarra**

Spain

**Rintze Zelle**

USA

**Xianhai Zeng**

China

**Zhanying Zhang**

Australia

**Chunyi Zhang**

China

**Yuqing Zhang**

China

**Bosen Zhang**

USA

**Haitao Zhang**

USA

**Taiying Zhang**

USA

**Y-H Percival Zhang**

USA

**Linguo Zhao**

China

**Xinqing Zhao**

China

**Zongbao Zhao**

China

**Huimin Zhao**

USA

**Pu Zheng**

China

**Chi Zhenming**

China

**Shungui Zhou**

China

**Zhi-Gang Zhou**

China

**Zhihua Zhou**

China

**Junyong ZHU**

USA

**Min-Hua Zong**

China

